# Supplementary material for: German validation of three ethics questionnaires: Consequentialist scale, ethical standards of judgment questionnaire, and revised ethics position questionnaire
Source: PLoS One. 2025 May 2;20(5):e0319937. doi: 10.1371/journal.pone.0319937 (PMC12047776; doi:10.1371/journal.pone.0319937)
Supplement: S2 Appendix — (PDF) [file pone.0319937.s002.pdf]

Consequentialist Scale (Robinson, 2012)

Unten finden Sie eine Reihe von allgemeinen Aussagen aufgelistet. Sie werden wahrscheinlich einigen Aussagen widersprechen und anderen zustimmen. Wir interessieren uns dafür, inwieweit Sie solchen Aussagen zustimmen oder widersprechen. Bitte lesen Sie jede Aussage sorgfältig durch und geben Sie für jede eine Antwort an (stimme überhaupt nicht zu, stimme eher nicht zu, neutral, stimme eher zu, stimme vollkommen zu):

1. Einige Regeln sollten niemals gebrochen werden.

2. Wenn Regeln und Gesetze das Glück der Menschen nicht maximieren, sollten sie ignoriert werden.

3. Es ist niemals moralisch gerechtfertigt, einem anderen Schaden zuzufügen.

4. Regeln und Gesetze müssen nur dann befolgt werden, wenn sie das Glück maximieren.

5. Wenn eine Handlung gegen die Grundregeln einer Gesellschaft verstößt, sollte sie nicht begangen werden, auch wenn sie viel Gutes bewirkt.

6. Einige Aspekte des menschlichen Daseins sind heilig und sollten niemals verletzt werden, egal wie groß der mögliche Gewinn ist.

7. Regeln und Gesetze spielen keine Rolle. Nur die Frage, ob eine Handlung Glück bringt, zählt bei der Entscheidung, wie gehandelt werden soll.

8. Einige Regeln und Gesetze sind universell und bindend, unabhängig von den Umständen, in denen man sich befindet.

9. Menschen, denen es nicht gelingt, ihr Glück zu maximieren, handeln moralisch falsch.

10. Das einzige moralische Prinzip, das befolgt werden muss, ist die Maximierung des Glücks.

The Ethical Standards of Judgement Questionnaire (Love, Salinas & Rotman, 2020)

Bitte lesen Sie auch die nachfolgenden Aussagen sorgfältig durch und geben Sie für jede eine Antwort an (stimme überhaupt nicht zu, stimme eher nicht zu, neutral, stimme eher zu, stimme vollkommen zu):

1. Wenn sich Menschen in ethischen Fragen nicht einig sind, bemühe ich mich um tragfähige Kompromisse.

2. Wenn ich über ethische Probleme nachdenke, versuche ich, praktische, umsetzbare Alternativen zu entwickeln.

3. In einer sich verändernden Welt ist es wichtig, dass Gesellschaften auf neue Bedingungen reagieren und sich an diese anpassen.

4. Lösungen für ethische Probleme werden in der Regel als Schattierungen von Grau angesehen.

5. Bei ethischen Entscheidungen sollte man die Bedürfnisse, Ansprüche und Anliegen anderer Menschen berücksichtigen.

6. Das Ziel der Regierung sollte es sein, das bestmögliche Leben für ihre Bürger und Bürgerinnen zu fördern.

7. Lösungen für ethische Probleme sind meist schwarz-weiß.

8. Handlungen einer Person sollten als richtig oder falsch beschrieben werden.

9. Eine Nation sollte ihrem Erbe und ihren Wurzeln die größte Aufmerksamkeit schenken.

10. Gesellschaften sollten stabilen Traditionen folgen und eine unverwechselbare Identität bewahren.

11. Eine Lüge zu äußern ist falsch, weil es für niemanden richtig wäre, zu lügen.

12. Unmoralisches Verhalten lässt sich am besten als Verstoß gegen einen Rechtsgrundsatz beschreiben.

Ethics Position Questionnaire (EPQ-5) (O'Boyle & Forsyth, 2021)

Bitte lesen Sie sich sorgfältig die folgenden zehn Aussagen durch und geben Sie an, ob Sie diesen zustimmen oder nicht (stimme überhaupt nicht zu, stimme eher nicht zu, neutral, stimme eher zu, stimme vollkommen zu).

1. Man sollte sicherstellen, mit seinen Handlungen niemals absichtlich einem anderen zu schaden, auch nicht in geringem Maße.

2. Potenzielle Schädigungen Dritter in Kauf zu nehmen, ist immer falsch, egal welche Vorteile erzielt werden.

3. Man sollte niemals eine andere Person psychisch oder physisch verletzen.

4. Man sollte keine Handlung ausführen, die in irgendeiner Weise die Würde und das Wohl eines anderen Menschen bedrohen könnte.

5. Wenn eine Handlung einem unschuldigen Anderen schaden könnte, sollte sie unterlassen werden.

6. Was moralisch ist, variiert zwischen Situationen und Gesellschaften/Kulturen.

7. Moralische Standards sollten als etwas Individuelles angesehen werden; Was eine Person als moralisch betrachtet, kann eine andere Person als unmoralisch bewerten.

8. Die Frage, was moralisch ist, kann niemals beantwortet werden, da die Entscheidung, was moralisch oder unmoralisch ist, individuell ist.

9. Moralische Standards sind einfach persönliche Regeln, die angeben, wie sich eine Person verhalten sollte. Sie sollten nicht verwendet werden, um über andere zu urteilen.

10. In zwischenmenschlichen Beziehungen sind ethische Probleme so komplex, dass es Personen erlaubt sein sollte, ihre eigenen persönlichen Regeln zu formulieren.
