# Supplementary material for: German validation of three ethics questionnaires: Consequentialist scale, ethical standards of judgment questionnaire, and revised ethics position questionnaire
Source: PLoS One. 2025 May 2;20(5):e0319937. doi: 10.1371/journal.pone.0319937 (PMC12047776; doi:10.1371/journal.pone.0319937)
Supplement: S3 Appendix — (PDF) [file pone.0319937.s003.pdf]

Consequentialist Scale inter-item correlations

	CS1	CS2	CS3	CS4
CS1	1,00	-0,16	0,13	-0,18
p*	.	0,01	0,04	0,00
CS2	-0,16	1,00	-0,02	0,55
p	0,01	.	0,79	<.001
CS3	0,13	-0,02	1,00	-0,04
p	0,04	0,79	.	0,56
CS4	-0,18	0,55	-0,04	1,00
p	0,00	<.001	0,56	.
CS5	0,19	-0,14	0,29	-0,09
p	0,00	0,03	<.001	0,16
CS6	0,23	-0,14	0,23	-0,23
p	<.001	0,03	<.001	<.001
CS7	-0,18	0,36	0,01	0,54
p	0,00	<.001	0,88	<.001
CS8	0,44	-0,14	0,22	-0,27
p	<.001	0,02	<.001	<.001
CS9	-0,03	0,18	0,09	0,26
p	0,63	0,00	0,15	<.001
CS10	-0,18	0,33	0,03	0,40
p	0,00	<.001	0,60	<.001

*p-value for significance of the correlation

ESJQ inter-item correlations

	ESJQ1	ESJQ2	ESJQ3	ESJQ4	ESJQ5
ESJQ1	1,00	0,43	0,15	0,07	0,19
p*	.	<.001	0,02	0,30	0,00
ESJQ2	0,43	1,00	0,20	0,10	0,11
p	<.001	.	0,00	0,10	0,09
ESJQ3	0,15	0,20	1,00	0,18	0,35
p	0,02	0,00	.	0,00	<.001
ESJQ4	0,07	0,10	0,18	1,00	0,13
p	0,30	0,10	0,00	.	0,03
ESJQ5	0,19	0,11	0,35	0,13	1,00
p	0,00	0,09	<.001	0,03	.
ESJQ6	0,09	0,14	0,19	0,14	0,20
p	0,14	0,03	0,00	0,02	0,00
ESJQ7	-0,11	-0,08	-0,25	-0,22	-0,27
p	0,08	0,19	<.001	<.001	<.001
ESJQ8	-0,03	-0,04	-0,12	0,04	-0,02
p	0,67	0,55	0,06	0,51	0,77
ESJQ9	0,07	0,04	-0,23	-0,16	-0,16
p	0,24	0,55	<.001	0,01	0,01

ESJQ10	0,07	-0,02	-0,31	-0,23	-0,20
p	0,25	0,75	<.001	<.001	0,00
ESJQ11	0,01	0,07	-0,14	-0,18	-0,05
p	0,83	0,26	0,03	0,00	0,43
ESJQ12	0,07	0,08	-0,13	-0,06	-0,12
p	0,29	0,17	0,04	0,38	0,04

*p-value for significance of the correlation

EPQ-5 inter-item correlations

	EPQ5_1	EPQ5_2	EPQ5_3	EPQ5_4	EPQ5_5
EPQ5_1	1,00	0,61	0,55	0,54	0,57
p*	.	<.001	<.001	<.001	<.001
EPQ5_2	0,61	1,00	0,54	0,52	0,50
p	<.001	.	<.001	<.001	<.001
EPQ5_3	0,55	0,54	1,00	0,67	0,50
p	<.001	<.001	.	<.001	<.001
EPQ5_4	0,54	0,52	0,67	1,00	0,58
p	<.001	<.001	<.001	.	<.001
EPQ5_5	0,57	0,50	0,50	0,58	1,00
p	<.001	<.001	<.001	<.001	.
EPQ5_6	-0,08	-0,14	-0,15	-0,10	-0,01
p	0,22	0,02	0,02	0,11	0,82
EPQ5_7	-0,12	-0,04	-0,08	-0,11	-0,02
p	0,06	0,51	0,22	0,08	0,79
EPQ5_8	-0,08	0,01	-0,01	-0,11	0,07
p	0,18	0,86	0,90	0,08	0,23
EPQ5_9	0,04	0,05	0,07	-0,04	0,04
p	0,53	0,43	0,24	0,50	0,57
EPQ5_10	-0,01	-0,01	-0,03	-0,05	-0,01
p	0,82	0,85	0,65	0,42	0,86

*p-value for significance of the correlation

CS5	CS6	CS7	CS8	CS9	CS10
0,19	0,23	-0,18	0,44	-0,03	-0,18
0,00	<.001	0,00	<.001	0,63	0,00
-0,14	-0,14	0,36	-0,14	0,18	0,33
0,03	0,03	<.001	0,02	0,00	<.001
0,29	0,23	0,01	0,22	0,09	0,03
<.001	<.001	0,88	<.001	0,15	0,60
-0,09	-0,23	0,54	-0,27	0,26	0,40
0,16	<.001	<.001	<.001	<.001	<.001
1,00	0,24	-0,14	0,17	0,10	0,01
.	<.001	0,02	0,01	0,09	0,83
0,24	1,00	-0,20	0,36	-0,05	-0,07
<.001	.	0,00	<.001	0,40	0,24
-0,14	-0,20	1,00	-0,30	0,27	0,48
0,02	0,00	.	<.001	<.001	<.001
0,17	0,36	-0,30	1,00	-0,02	-0,12
0,01	<.001	<.001	.	0,70	0,05
0,10	-0,05	0,27	-0,02	1,00	0,48
0,09	0,40	<.001	0,70	.	<.001
0,01	-0,07	0,48	-0,12	0,48	1,00
0,83	0,24	<.001	0,05	<.001	.

ESJQ6	ESJQ7	ESJQ8	ESJQ9	ESJQ10	ESJQ11	ESJQ12
0,09	-0,11	-0,03	0,07	0,07	0,01	0,07
0,14	0,08	0,67	0,24	0,25	0,83	0,29
0,14	-0,08	-0,04	0,04	-0,02	0,07	0,08
0,03	0,19	0,55	0,55	0,75	0,26	0,17
0,19	-0,25	-0,12	-0,23	-0,31	-0,14	-0,13
0,00	<.001	0,06	<.001	<.001	0,03	0,04
0,14	-0,22	0,04	-0,16	-0,23	-0,18	-0,06
0,02	<.001	0,51	0,01	<.001	0,00	0,38
0,20	-0,27	-0,02	-0,16	-0,20	-0,05	-0,12
0,00	<.001	0,77	0,01	0,00	0,43	0,04
1,00	0,09	0,07	-0,04	-0,01	0,11	0,05
.	0,16	0,27	0,53	0,89	0,07	0,38
0,09	1,00	0,32	0,08	0,10	0,07	0,17
0,16	.	<.001	0,21	0,10	0,26	0,01
0,07	0,32	1,00	0,15	0,18	0,17	0,22
0,27	<.001	.	0,02	0,00	0,01	<.001
-0,04	0,08	0,15	1,00	0,59	0,20	0,25
0,53	0,21	0,02	.	<.001	<.001	<.001

-0,01	0,10	0,18	0,59	1,00	0,30	0,32
0,89	0,10	0,00	<.001	.	<.001	<.001
0,11	0,07	0,17	0,20	0,30	1,00	0,36
0,07	0,26	0,01	<.001	<.001	.	<.001
0,05	0,17	0,22	0,25	0,32	0,36	1,00
0,38	0,01	<.001	<.001	<.001	<.001	.

EPQ5_6	EPQ5_7	EPQ5_8	EPQ5_9	EPQ5_10
-0,08	-0,12	-0,08	0,04	-0,01
0,22	0,06	0,18	0,53	0,82
-0,14	-0,04	0,01	0,05	-0,01
0,02	0,51	0,86	0,43	0,85
-0,15	-0,08	-0,01	0,07	-0,03
0,02	0,22	0,90	0,24	0,65
-0,10	-0,11	-0,11	-0,04	-0,05
0,11	0,08	0,08	0,50	0,42
-0,01	-0,02	0,07	0,04	-0,01
0,82	0,79	0,23	0,57	0,86
1,00	0,39	0,38	0,25	0,24
.	<.001	<.001	<.001	<.001
0,39	1,00	0,75	0,42	0,32
<.001	.	<.001	<.001	<.001
0,38	0,75	1,00	0,50	0,38
<.001	<.001	.	<.001	<.001
0,25	0,42	0,50	1,00	0,38
<.001	<.001	<.001	.	<.001
0,24	0,32	0,38	0,38	1,00
<.001	<.001	<.001	<.001	.
